# Predicting postoperative complications after bariatric surgery: the Bariatric Surgery Index for Complications, BASIC

**DOI:** 10.1007/s00464-017-5494-0

**Published:** 2017-03-31

**Authors:** Usha K. Coblijn, Julian Karres, Christel A. L. de Raaff, Steve M. M. de Castro, Sjoerd M. Lagarde, Willem F. van Tets, H. Jaap Bonjer, Bart A. van Wagensveld

**Affiliations:** 1 Departments of Surgery, Onze Lieve Vrouwe Gasthuis, locatie West (Prevoiusly Sint Lucas Andreas Ziekenhuis, Amsterdam), Jan Tooropstraat 161, 1064 AE Amsterdam, The Netherlands; 2 Departments of Surgery, Tergooi Ziekenhuizen, Blaricum, The Netherlands; 3 Departments of Surgery, Onze Lieve Vrouwe Gasthuis, Amsterdam, The Netherlands; 4 Departments of Surgery, Erasmus Medisch Centrum, Rotterdam, The Netherlands; 5 Departments of Surgery, Vrije Universiteit Medisch Centrum, Amsterdam, The Netherlands

**Keywords:** Bariatric surgery, Complications, Prediction model, Roux-en-Y gastric bypass, Sleeve gastrectomy

## Abstract

**Background:**

Around 20% of bariatric surgery patients develop a short- or long-term complication.

**Objective:**

Aim of this study was to develop a risk model predicting complications: the Bariatric Surgery Index for Complications (BASIC).

**Setting:**

The Obesity Center Amsterdam, located in a large teaching hospital, in Amsterdam, The Netherlands.

**Methods:**

A prospective consecutive database including patients operated between November 2007 and February 2015 was used. For the BASIC, analysis according to the TRIPOD statement was performed to identify risk factors for complications. Class I included patients with zero to one risk factor, class II patients with two risk factors, and class III patients with three or more risk factors.

**Results:**

Of 1709 analyzed patients, mean age was 45 years (±SD 10.7), 1393 (81.5%) were female; mean body mass index was 44.5 kg/m^2^ (6.8). Overall, 271 (15.9%) patients developed a complication of which 197 (72.5%) occurred within 30 days. Predictors in multivariable analysis were use of anticoagulants (odd’s ratio (OR) 1.5); chronic obstructive pulmonary disease (OR 2.3); dyslipidemia (OR 1.4); gender (OR 1.4); psychiatric history (OR 1.3); and revisional surgery (OR 1.5). In class I, 13.5% (181 out of 1338) experienced complications, in class II 58 (21.6%) of the 269 patients and in class III 32 (31.4%) of the 102 patients, respectively. There was a significant difference (*p* < *0.001*) in both overall and 30 day complications.

**Conclusion:**

The BASIC uses six preoperative variables to classify patients in a low-, intermediate-, or high-risk group for postoperative complications after bariatric surgery.

Obesity is a major health problem and the incidence is increasing worldwide. So far, the only treatment for morbid obesity with good long-term results is bariatric surgery. Recently, there is a shift from laparoscopic adjustable gastric banding (LAGB) to laparoscopic Roux-en Y gastric bypass (LRYGB) and laparoscopic sleeve gastrectomy (LSG). At present, about 49% of procedures are LRYGB [1]. This procedure has become relatively safe with acceptable morbidity and mortality [2, 3].

As a predictive tool for postoperative mortality, the obesity surgery mortality risk score (OS-MRS) can be used [4, 5]. This score consists of the following parameters: body mass index (BMI) ≥ 50 kg/m^2^; age ≥ 45; male gender; hypertension; risk of pulmonary embolism. Although it is unknown if the OS-MRS accurately predicts the risk for postoperative complications, it is sometimes used for that purpose [6, 7].

A common method to inventory postoperative complications is the Clavien-Dindo classification [8], which is based on the required intervention following complications and ranges from no intervention, reoperation, or radiological intervention, intensive care unit admission, and death. After laparoscopic bariatric surgery, the incidence and severity of short-term (within 30 days postoperatively) adverse events vary between the 4.9 and 10% [9–11]. Severe complications such as anastomotic/ staple line leakage, stenosis or stricture of the anastomosis and pulmonary embolism occur in 3% of the patients; death in 0.2% [6, 12]. Preoperative risk assessment is one of the most important components of surgical decision making. A risk assessment system for bariatric surgery should provide an accurate representation of the complication risk using only the information that is preoperatively available The aim of this study is to develop the Bariatric Surgery Index for Complications (BASIC), a simple, adequate scoring system, similar to the OS-MRS based on preoperative parameters, assessing the risk on postoperative complications.

## Methods

### Study cohort

All patients who underwent primary or revisional LRYGB or LSG or pouch revision of previous LRYGB from November 2007 onwards were prospectively entered in a consecutive database. Patients operated until January 2014 were included in the analysis, as they had a minimal follow-up of 12 months. Patients were selected according to their surgical procedure. Included were those who require a staple line or anastomosis as all these kind of surgeries contain the risk of anastomotic or staple line leakage. Patients who underwent laparoscopic gastric banding or banded bypass as secondary stage procedure were excluded from analysis. Patients eligibility regarding bariatric surgery was screened according to the IFSO guidelines [13].

All patients at our center receive a routine preoperative screening by a multidisciplinary team including a medical doctor, prior to surgery. All patients are interviewed concerning their medical history and drug usage, a full physical examination is performed and no patient is operated without information of their general practitioner. If the suspicion of any disease (e.g., diabetes, dyslipidemia) or insufficient treatment of existing disease exist patients are subjected to the appropriate diagnostics before they can proceed to surgery. Screening for obstructive sleep apnea (by poly(somno)graphy), hypertension, the presence of H. Pylori [with oesophagogastroduodenoscopy and CLO test (prior to July 2014) or feces test (after July 2014)] and psychopathology by means of a consult with a psychotherapist and usage of questionnaires is mandatory in all patients prior to surgery.

The required data for this risk prediction model were collected from the database. Patient characteristics, such as age, gender, comorbidities, BMI, and operative data were retrieved. Complications were scored by both type and severity using the Clavien-Dindo classification with the following endpoints: mortality; multi-organ failure; single organ failure; surgical intervention; radiological intervention; medical treatment; no intervention needed; and no complications. A Clavien-Dindo classification of three points or higher was regarded as a severe complication. The investigators, collecting the data for the database, based on the electronic patient status, assigned the Clavien-Dindo classification points. In addition to this classification, the nature of the complication was scored as well as if it were short-term (occurring within 30 days after surgery) or long-term complications. A complication graded Clavien-Dindo 3 or higher was considered a serious complication, those were also separately analyzed in the risk stratification.

This study has been approved by the local medical ethics committee; no individual informed consent was necessary as it was a retrospective analysis.

Twenty-four patient variables within the database were regarded as possible risk factors and subsequently analyzed for postoperative complications (Appendix Table 8). COPD was defined as being diagnosed by a pulmonary doctor with at least COPD GOLD II; diabetes was divided in patients with and without insulin usage; dyslipidemia was scored when patients used cholesterol lowering drugs or were diagnosed with dyslipidemia by means of a blood test; OSA was diagnosed in every patient by means of a poly(somno)graphy; a psychiatric disease was diagnosed when patients used psychiatric drugs (including anti-depressants) and/or went through extensive therapy; all gastric disorders were found by the performance of a gastroscopy preoperatively; the usage of NSAID’s and corticosteroids were based on medication history taking and finally the usage of anticoagulants was defined as the chronic usage of any anticoagulant including platelet inhibitors. Variables with more than 5% missing data were discarded after sub-analysis showed no effect of these variables. For the prediction of complications, three categories were distinguished with a uni- and multivariable regression analysis: class I included patients who had a maximum of one risk factor, class II comprised patients with two risk factors and class III was with patients with three or more risk factors.

### Surgical procedure

All procedures were carried out by four experienced bariatric surgeons or under their direct supervision. The procedures were performed as previous described [14]. If patients underwent a revisional procedure from an adjustable gastric band, it started with removal of the band followed by direct revision, after which the Port-a-cath was removed prior to skin closure.

For all procedures pneumoperitoneum was obtained. Five trocars (three 12 mm and two 5 mm) were used. In case of LRYGB, the pouch was formed with one horizontal and 3–4 vertical firings of a 45 mm endoscopic stapler (Johnson and Johnson, Sommerville, NY, USA) in the lesser curvature of the stomach. Subsequently the gastrojejunostomy was created in an antecolic, antegastric fashion, posterior by means of a stapler and anteriorly hand sewn using a barbed suture V-loc™ (Covidien, Dublin, Ireland). This was followed by the jejunojejunostomy at 120–150 cm and transection of the connecting loop.

The LSG was created using multiple firings of the Echelon 60 endoscopic stapler. The remnant stomach was removed through the most lateral 12 mm port after the trocar was removed and the incision enlarged (2–3 cm) and sent for pathologic examination.

Pouch revision was performed after inspection in the same fashion as creation of the pouch at primary LRYGB. Often the anastomosis was revised.

### Statistical analysis

All data were analyzed using SPSS version 21.0 for windows (SPSS Chicago, IL, USA).

For the BASIC, uni- and multivariable regression analyses were performed to identify the variables predicting complications. Multivariable regression analysis was performed according to the TRIPOD statement, which accepts *p* values up to 0.157 to enhance the applicability of the model to other patient groups [15]. After classifying the patients, the Chi-square test was used to demonstrate any statistical significance, of which definition was set at a two sided *p* value of <0.05. Select cases was used to detect the highest association between groups after which correction for multiple testing took place.

## Results

From November 2007 till February 2015, a total of 1709 patients underwent bariatric surgery.

Most patients, 1393 (81.5%) were female; the mean age was 44.6 years (SD 10.7) and the mean BMI was 44.5 kg/m^2^ (SD 6.8). Baseline characteristics are displayed in Table 1.

**Table 1:** Baseline characteristics

Baseline	No complication	Complication	*p* value
Age (years) (SD)	44.4 (10.7)	45.9 (10.7)	**0.035**
BMI (kg/m^2^) (SD)	44.6 (6.7)	43.8 (7.1)	0.097
Gender (F/M)	1183/255	210/61	0.063
Diabetes (%)	403 (28.0)	87 (32.1)	0.175
Dyslipidemia (%)	317 (22.1)	81 (30.1)	**0.004**
Hypertension (%)	572 (39.8)	130 (48.0)	**0.012**
Revisional surgery (%)	249 (17.3)	68 (25.1)	**0.003**

Bold values indicate statistical analysis at *p* < 0.05

*BMI* body mass index, *F* female, *M* male

Primary LRYGB was performed in 1283 patients (75.1%), followed by revision from LAGB into LRYGB in 281 patients (16.4%) (Table 2).

**Table 2:** Type of surgery

Procedure	Total number	Percentage
LRYGB	1283	75.1
LSG	109	6.4
LSG to LRYGB	15	0.9
LAGB to LRYGB	281	16.4
LAGB to LSG	11	0.6
Mason to LRYGB	1	0.1
Mason to LSG	1	0.1
Pouch revision	8	0.5
Total procedures	1709	100

*LRYGB* laparoscopic Roux en Y gastric bypass, *LSG* laparoscopic sleeve gastrectomy *LAGB* laparoscopic adjustable gastric band

Overall, postoperative complications occurred in 271 patients (15.9%) of which 197 (72.7%) were short-term complications, (Table 3). Twenty-two patients had a leakage of the gastrojejunostomy (GJS), 42 patients suffered from (severe) peri- or postoperative bleeding, 15 patients had a stenosis of the GJS, and ten patients developed an internal herniation approximately 1 year after surgery. Of all 271 complications, 140 patients (8.2% of 1709) had a severe complication according to the Clavien-Dindo classification. Five patients (0.3%) died (Clavien-Dindo 5), three of them had revisional surgery and subsequently died of cardiac tamponade, pulmonary embolism, and bowel strangulation, respectively, two patients died of sepsis and multi-organ failure after anastomotic leakage from primary LRYGB.

**Table 3:** Number of complications

Complication	Total number	Percentage
Short-term	197	11.5
Overall	271	15.9
Clavien-Dindo ≥ 3	140	8.2

### Risk analysis

Twenty-four preoperative variables were considered in the univariate analysis as a risk predictor whereof five were significant *p* < 0.05: age of 60 years and above; hypertension; dyslipidemia; chronic obstructive pulmonary disease (COPD); and revision from previous bariatric surgery. Use of anticoagulants and a history of psychiatric diseases were added according to the TRIPOD statement. In the multivariable analysis, backwards selection resulted in elimination of diabetes type II; followed by age above 60; alcohol; corticosteroids; BMI above 50; gastroesophageal reflux disease; smoking; NSAID’s; cholecystectomy; hypertension and history of trombo-embolic event respectively. Consequently, anticoagulant usage; COPD; dyslipidemia; gender; psychiatric history, and revisional surgery provided the most optimal multivariable model as displayed in Table 4. As all factors had an odd’s ratio between the 1.3 and 2.3, one point was assigned to each of the contributing factors.

**Table 4:** Multivariable analysis, risk factors BASIC

Variable	*p* value	Odd’s ratio	95% CI for the Exp.	
Anticoagulants	0.142	1.454	0.883	2.394
COPD	0.007	2.271	1.254	4.113
Dyslipidemia	0.042	1.396	1.012	1.928
Gender (male)	0.037	1.438	1.023	2.023
Psychiatric history	0.137	1.298	0.921	1.831
Revisional surgery	0.021	1.498	1.064	2.110

*COPD* chronic obstructive pulmonary disease

### Risk classification

Patients were divided in classes using the variables according to the multivariable analysis and the description in the “methods” section. A differentiation was made between short-term and overall complications. The overall complication analysis showed the following results: class I existed of 1338 (78.3%) patients of which 181 (13.5%) suffered from a complication, class II comprised 269 patients (15.7%) of which 142 (21.6%) patients had a complication and class III existed of 102 patients (6.0%) of which 32 (31.4%) developed a complication. The difference in incidence of complications between the three classes was statistically significant with a *p* value of 0.001 (Table 5).

Not only was this significant in the occurrence of overall complications but also within the patient group who developed a short-term complication (*p* = 0.001). These short-term complications occurred in 136 (10.2%) of the patients in class I, 40 (14.9%) patients in class II, and 21 (20.6%) patients in class III, respectively (Table 6).

**Table 5:** Distribution among classes over all complications

Classification	Total number (%)	Complication (%)	*p* value
Class I (0–1 risk factor)	1338 (78.3)	181 (13.5)	**0.001**
Class II (2 risk factors)	269 (15.7)	58 (21.6)	
Class III (≥3 risk factors)	102 (6.0)	32 (31.4)	

Bold value indicates statistical analysis at *p* < 0.05

**Table 6:** Distribution among classes short-term complications

Classification	Total number (%)	Complication (%)	*p* value
Class I (0–1 risk factor)	1338 (78.3)	136 (10.2)	**0.001**
Class II (2 risk factors)	269 (15.7)	40 (14.9)	
Class III (≥3 risk factors)	102 (6.0)	21 (20.6)	

Bold value indicates statistical analysis at *p* < 0.05

The significant difference between classes was mainly caused by the difference between class I and III but the difference between all classes was significant in the overall complication rate. Analyzing short-term complications alone, the significance was mainly caused by comparison of class I and III, followed by I and II. No difference between II and III could be identified. When dividing complications in mild (Clavien-Dindo ≤ 3) and severe (Clavien-Dindo > 3) complications, a significant difference exists between class I versus III and class I versus II.

The results of this sub-analysis are displayed in Table 7.

**Table 7:** Select cases between groups

BASIC	Complication rate	*p* value	OR	CI
Short-term complications				
I vs. III	10.2 vs. 20.6	**0.001**	2.291	1.374–3.822
I vs. II	10.2 vs. 14.9	**0.024**	1.544	0.056–2.257
II vs. III	14.9 vs. 20.6	0.185	1.484	0.826–2.666
Overall complications				
I vs. III	13.5 vs. 31.4	<**0.001**	2.922	1.870–4.567
I vs. II	13.5 vs. 21.6	**0.001**	1.757	1.263–2.444
II vs. III	21.6 vs. 31.4	**0.049**	1.663	0.999–2.767

Severe complications				
I vs. III	7.2 vs. 15.3	**0.004**	2.330	1.292–4.202
I vs. II	7.2 vs. 13.0	**0.002**	1.931	1.270–2.935
II vs. III	13.0 vs. 15.3	0.575	1.207	0.625–2.329

Bold values indicate statistical analysis at *p* < 0.05

## Discussion

This study developed a risk model for postoperative complications in an attempt to predict the development of complications after bariatric surgery. The study identified six preoperative variables, which are all independent risk factors for the occurrence of postoperative complications. With this risk model, it is possible to select patients with a two times higher risk of postoperative complications compared to the general bariatric population.

It might seem odd that dyslipidemia was identified as a risk factor for postoperative complications. However, as one of the pillars of metabolic syndrome, patients with dyslipidemia are in less good condition or shape than patients without dyslipidemia. Furthermore the presence of dyslipidemia increases the likelihood of cardiovascular diseases and maybe also cardiovascular complications. For example, ischemia is thought to be a part in the pathophysiology of the development of anastomotic leakage after bariatric surgery or in marginal ulceration.

Numerous authors have attempted to predict the risk for complications after bariatric surgery. Some by adjusting or applying the OS-MRS to their population [6, 16], others by developing a new model based on national databases [17–19]. Gupta et al. provided a risk prediction model that calculates the risk of major postoperative complications. The model was based on the following variables; type of surgery, extremes of BMI 35 to <45 and >60 kg/m^2^, recent myocardial infarction, bleeding disorder, functional dependency in daily life, hypertension, and stroke. The complications were divided into 17 possibilities according to their nature or required intervention [12, 17]. BMI was also analyzed in the present study, as continuous and dichotomous variable but was not associated with complications. This might be due to the increased experience in large volume centers causing less influence of BMI on operative outcome. Therefore, concentrating bariatric surgery in large volume centers might be important to improve the results. Subsequently, Birkmeyer et al. found that surgical skill was strongly related to volume and not to clinical important differences as patient age, sex, or BMI. Obviously, they found a difference in surgical skill and complication rate; however, the present research was performed in one high-volume center, automatically eliminating the bias of different centers or surgeons who perform less procedures annually [20]. Due to increasing experience in bariatric procedures and high-volume centers, some previously described patient characteristics such as BMI will become less important causes in the development of postoperative complications, as demonstrated in the present study.

In contrast, functional dependency and a history of stroke were not separate variables in the present study and therefore not taken into consideration, which might be of additional value in validating this cohort or should be taken into account in future studies [12].

Although it is important to detect patients with an increased risk of severe long-term complication, it is difficult to predict this risk based on preoperative variables. Preoperative patient characteristics change as this is the primary focus of bariatric surgery with the main interest in decreasing patient’s comorbidities [2, 21].

One limitation of the present study is the relatively small sample size (*n* = 1709). Due to the low complication rate, detecting inter patient variability requires larger study populations.

Another limitation, partially caused by the same problem of a small sample size, is that this risk model predicts the risk on overall complications; however, it does not provide the risk factors for each complication on itself. For example, it is known is that smoking, NSAIDS, and corticosteroids increase the risk on marginal ulceration; however, these parameters did not influence the overall complication rate, possibly due to the fact that the influence of marginal ulceration in the total complication rate is small. Revisional surgery, as in the present cohort, increases the risk on postoperative complications by itself [9, 19]. It is advisable to focus in future, prospective studies, on the differences between patients in primary and revisional bariatric surgery, such as the possibility of malnutrition. Another, possible limitation is that the parameters collected in the database were determined prior to this study; all data available on the patients were entered preoperatively and all other perioperative and postoperative data were prospectively collected. This might induce the possibility that certain variables were not collected, which would influence the risk on complications. However, the objective of this study was to identify risk factors based on preoperative patient characteristics in an attempt to select patients with a higher risk preoperatively and if possible adjust the perioperative care for which this study was sufficient.

Finally, all variables predicting complications in the present cohort were equally treated in the present paper despite small differences in odd’s ratio’s to increase the usability of this BASIC prediction model in daily clinical practice.

Validation of this risk model in a larger cohort is necessary. As many predictors were assessed, it might be possible, although highly unlikely, that the present findings are a coincidence and an expression of the general poor health of these subjects analyzed as a cohort, but no significant risk factor in the individual patient. Moreover, validation of this model in a different cohort than its development is preferable since this increases the applicability of the model in other patient groups. The development of a prediction model based on a sufficient area under the curve with good calibration is preferred but not possible in this relatively small cohort. Larger cohort studies would provide the possibility to develop a prediction model with predictive property and more detailed discrimination between patients, possible thereby enhancing the general applicability of the model.

As a final remark, it is possible, even likely, that other risk factors, are not analyzed in this study, exist and thereby influence the patient outcomes.

Although laparoscopic bariatric surgery has a low complication rate, it is performed in a patient population with significant co-morbidity, even for elective surgery. Therefore, it is important to identify patients based on their own characteristics who have a high risk on postoperative complications. As bariatric centers become more high volume, care is increasingly adjusted to accommodate the bariatric surgery patients, therefore the inter-center variability will become less a confounder in predicting complications between those centers.

## Conclusion

The BASIC is based on six preoperative patient characteristics to classify patients in three risk classes: low-, intermediate-, and high-risk as class I, II, and III, respectively. This model provides the possibility to identify a small subgroup of patients with a two times higher (30.6%) risk of overall postoperative complications following bariatric surgery.

While as of now the BASIC lacks validation, the question can be raised if patients in class III with three or more risk factors should be selected for surgery according to the same eligibility criteria or treated perioperatively under the same conditions as patients from class I or II. Preoperative risk assessment can facilitate patient specific, adjusted care and lead to improved patient outcomes after bariatric surgery.

## Appendix

See Table 8.

**Table 8:** Overall complications, univariate analysis

Variable	Total no of patients	No of patients	Complication	*p* value	CI (95%)	
Age						
≥60	1709	155	30	0.214	0.857	1.991
<60		1553	125			
Gender						
F	1709	1393	210	0.064	0.9833	1.848
M		316	61			
BMI						
≥50	1707	337	48	0.360	0.610	1.197
<50		1370	289			
COPD						
Yes	1709	63	18	**0.006**	1.255	3.866
No		1646	45			
Diabetes						
Yes	1708	490	87	0.678	0.774	1.482
No		1281	403			
NIDDM						
Yes	1708	288	51	0.349	0.840	1.641
No		1420	237			
IDDM						
Yes	1708	209	37	0.438	0.794	1.703
No		1499	172			
Dyslipidemia						
Yes	1706	398	81	**0.004**	1.140	2.032
No		1308	317			
Hypertension						
Yes	1709	1007	130	**0.012**	1.076	1.812
No		702	572			
OSA Y/N						
Yes	1538	597	146	0.904	0.741	1.303
No		941	795			
Sever OSAS (AHI > 30)						
Yes	1529	305	51	0.542	0.792	1.559
No		1221	257			
Psychiatric disease						
Yes	1708	316	58	0.182	0.903	1.721
No		1391	258			
Trombo-embolic event						
Yes	1709	58	8	0.664	0.396	1.802
No		1651	50			
GERD						
Yes	1636	554	92	0.507	0.832	1.451
No		1082	462			
Gastritis						
Yes	1135	217	39	0.483	0.779	1.695
No		918	178			
Hiatal Hernia						
Yes	1140	210	31	0.500	0.570	1.316
No		930	179			
H.Pylori						
Yes	1193	246	34	0.413	0.566	1.236
No		947	212			
Alcohol						
Yes	1640	641	99	0.900	0.773	1.340
No		999	542			
Smoking						
Yes	1457	332	59	0.191	0.899	1.703
No		1325	273			
NSAIDS						
Yes	1704	125	21	0.776	0.659	1.749
No		1579	104			
Corticosteroids						
Yes	1704	110	22	0.226	0.830	2.196
No		1594	88			
Anticoagulants						
Yes	1704	121	26	0.083	9.49	2.355
No		1583	95			
Cholecystectomy						
Yes	1709	201	37	0.293	0.837	1.802
No		1508	164			
Revisional surgery						
Yes	1709	317	68	**0.003**	1.177	2.174
No		1392	249			

Bold values indicate statistical analysis at *p* < 0.05

*CI* confidence interval, *BMI* body mass index, *NIDDM* non-insulin depended diabetes mellitus, *IDDM* insulin depended diabetes mellitus, *OSAS* obstructive sleep apnea, *AHI* apnea hypopnea index, *GERD* gastroesophageal reflux disease *H. Pylori* helicobacter pylori, *COPD* chronic obstructive pulmonary disease, *NSAIDS* non-steroidal anti-inflammatory drugs
